# B-A Chromosome Translocations Possessing an A Centromere Partly Overcome the Root-Restricted Process of Chromosome Elimination in *Aegilops speltoides*

**DOI:** 10.3389/fcell.2022.875523

**Published:** 2022-03-28

**Authors:** Daiyan Li, Alevtina Ruban, Jörg Fuchs, Houyang Kang, Andreas Houben

**Affiliations:** ^1^ Leibniz Institute of Plant Genetics and Crop Plant Research (IPK), Gatersleben, Germany; ^2^ State Key Laboratory of Crop Gene Exploration and Utilization in Southwest China, Sichuan Agricultural University, Chengdu, China; ^3^ Triticeae Research Institute, Sichuan Agricultural University, Chengdu, China; ^4^ KWS SAAT SE & Co. KGaA, Einbeck, Germany

**Keywords:** Aegilops speltoides, supernumerary B chromosome, X-ray irradiation, B-A translocation chromosome, programmed chromosome elimination, micronuclei

## Abstract

Some eukaryotes exhibit dramatic genome size differences between cells of different organs, resulting from the programmed elimination of chromosomes. *Aegilops speltoides* is an annual diploid species from the Poaceae family, with a maximum number of eight B chromosomes (Bs) in addition to its inherent seven pairs of standard A chromosomes (As). The Bs of this species undergo precise elimination in roots early in embryo development. In areal parts of the plant, the number of Bs is stable. To affect the root restricted process of B chromosome elimination, we employed X-ray mutagenesis, and different types of restructured Bs were identified. Standard Bs were observed in all analyzed shoots of mutagenized plants, while B-A translocations were only observed in 35.7% of F_1_ plants. In total 40 different B variants inconsistently escaped the elimination process in roots. As a result, mosaicism of B chromosome variants was found in roots. Only a small B chromosome fragment fused to an A chromosome was stably maintained in roots and shoots across F_1_ to F_3_ generations. The absence of B-A translocation chromosomes possessing a derived B centromere in root cells implies that the centromere of the B is a key component of the chromosome elimination process.

## Introduction

Not necessarily all somatic cells of an organism contain the same chromosomes (reviewed in Wang and Davis, 2014). In some eukaryotes, B chromosomes are present in generative tissue but absent in somatic tissue (listed in Jones and Rees, 1982). B chromosomes (Bs) are dispensable genome components of thousands of eukaryotic species, including plants, animals and fungi. Although supernumerary, many of them are preferentially inherited relative to the basic A chromosome (A) set (Jones and Rees, 1982). The maximal number of Bs the host can tolerate varies across species and is balanced by the B chromosomal drive and detrimental effects to the host associated with the presence of Bs. Whereas low copy numbers of Bs have no discernable impact on the host development, a high number of Bs may cause negative effects, especially on the growth vigour and fertility (Bougourd and Jones, 1997).

*Ae. speltoides* Tausch (SS genome) is an annual diploid species from the Poaceae family and is likely the wild progenitor of the B genome of allopolyploid wheat (Marcussen et al., 2014). In addition to its seven pairs of standard A chromosomes, this species carries up to eight B chromosomes (Kimber and Feldmann, 1987; Raskina et al., 2011). Reciprocal crosses between 0B and +B plants indicated that the drive of Bs occurs during the first mitosis in the male gametophyte whereas there is no accumulation of Bs during the development of female gametophytes (Mendelson and Zohary, 1972). Independent of the number of Bs present in the mother plant, in >93% of the first pollen grain mitoses Bs are preferentially transmitted to the generative nuclei *via* direct nondisjunction (Wu et al., 2019). A similar chromosome accumulation process is known for the B chromosome of rye (Hasegawa, 1934; Banaei-Moghaddam et al., 2012). Segregation analyses of induced and spontaneously arisen rye B chromosome variants resulted in the identification of the distal region of the long arm as the region controlling *in trans* the process of nondisjunction (Müntzing, 1945; Müntzing, 1946; Müntzing, 1948; Håkanson, 1959; Lima-De-Faria, 1962; Endo et al., 2008). It is unknown whether a comparable chromosome region controls the process of B chromosome drive in *Ae. speltoides*.

Unlike in rye, the Bs of *Ae. speltoides* are entirely absent in the roots but are stably present in aerial tissues (Mendelson and Zohary, 1972; Ohta, 1995; Ruban et al., 2014; Ruban et al., 2020). The elimination of B chromosomes is a strictly controlled and highly efficient root-specific process. B chromosomes undergo complete elimination in proto-root cells at the onset of embryo differentiation. Independent of centromere activity, B chromosomes demonstrate nondisjunction of chromatids and lagging in anaphase, resulting in micronucleation and subsequent elimination. This process might allow root tissues to survive the detrimental expression or overexpression of B chromosome-located root-specific genes with paralogs located on standard A chromosomes (Boudichevskaia et al., 2020; Ruban et al., 2020).

Here, we tested whether plants with root- and shoot-located B chromosomes could be generated by mutagenesis. Mutagenized *Ae. speltoides* plants were generated through pollinating non-irradiated 0B plants with X-ray irradiated + B pollen. In maize, Roman (1947), Liu et al. (2020) employed a similar mutagenesis strategy and produced B-A translocation lines and a number of B chromosome variants. Furthermore, a great variety of chromosomal types were produced by selecting crossover derivatives after crossing appropriate reciprocal A translocations with B-A translocations (Rakha and Robertson, 1970). These B-A translocations facilitated genetic studies to locate genes to specific chromosomal arms/segments and map restriction fragment length polymorphism (RFLP) loci (Beckett, 1978, 1991; Weber and Helentjaris, 1989). In rye, none of the generated B-A translocations was transmitted to the next generation (Hasterok et al., 2002), suggesting that B-A translocations are not heritable in this species. Besides irradiation, rearranged rye Bs in the background of wheat were produced by the application of a gametocidal system (Endo et al., 2008).

We systematically screened *de novo* generated *Ae. speltoides* mutant plants for B chromosome variants in shoots and roots by multicolor fluorescent *in situ* hybridization. Our analysis demonstrated that some B-A translocation chromosomes and other B chromosome variants inconsistently escaped the root-restricted chromosome elimination process in Ae*. speltoides.* Only a small fragment of a B chromosome arm fused to an A chromosome consistently evaded the elimination process.

## Materials and Methods

### Plant Material, Cultivation and Irradiation

*Aegilops speltoides* Tausch plants with and without B chromosomes from the Ramat Hanadiv population, Israel [accession 2-46, Institute of Evolution collection, Haifa, Israel (Belyayev and Raskina, 2013)] were used. All plants were cultivated under greenhouse conditions (16 h light, day temperature 20–24°C, night temperature 17–19°C) at the IPK (Gatersleben, Germany). At the stage of 3 or 4 leaves, plants were transferred to 12–15°C for 4 weeks to ensure better tillering and synchronous flowering. The whole spikes shortly before anthesis from plants carrying Bs were irradiated using an X-ray apparatus (Yxlon International, Hamburg) equipped with control panel MGC 41, high voltage generator MGP 40 and X-ray tube KB 150/6, with doses at 13, 15 and 18 Gray. After irradiation, pollen was collected and used to pollinate the plants without Bs. The resulting F_1_ plants and their selfing progenies (F_2_ and F_3_) were screened for the presence of rearranged B chromosomes by means of fluorescent *in situ* hybridization (FISH).

### B Chromosome Number Estimation by Flow Cytometry

The number of B chromosomes in plants used for irradiation experiment was calculated based on estimations of the DNA content by flow cytometry (Ruban et al., 2014). For this the DNA content of each plant was measured on a CyFlow Space (Partec) using *Secale cereale* L. (16.19 pg/2C; Genebank Gatersleben, accession number R 737) as an internal reference standard (Galbraith et al., 1983). The number of Bs per plant was then calculated based on the difference of DNA content between 0B plants and +B plants assuming a size 570 Mbp per B (Ruban et al., 2014).

### Chromosome Preparation

Material was collected from the plants grown in the greenhouse or from the seeds germinated at 25°C for several days in Petri dishes lined with two layers of wet filter paper. Chromosomes were prepared according to the protocol described by Komuro et al. (2013). In brief, root tips were cut when roots reached 1–1.5 cm either from seedlings or developed plants, while shoots were isolated when plants formed several tillers. The collected root tips and shoots were treated with ice-cold water for 24–26 h and then incubated in 90% acetic acid for 5 min, washed twice with ddH_2_O for 2 min each, followed by enzyme (2.5% pectinase, 2.5% pectolyase Y-23 and 2.5% cellulase R-10 dissolved in citrate buffer) digestion for 50–60 min at 37°C. After that, digested samples were washed by ddH_2_O, followed by 70% ethanol twice, then transferred to 100% acetic acid and were dropped on slides for FISH as described by Badaeva et al. (2018).

### Fluorescence *in Situ* Hybridization

The *Ae. speltoides* B-specific sequences AesTR-183, AesTR-205 (Wu et al., 2019), the (peri)centromere-specific sequence pBs301 (Cheng and Murata, 2003) and the A chromosome-specific repetitive sequence pSc119.2 (Tang et al., 2014) were used as FISH probes following the method described by Ruban et al. (2020) with minor modifications. Slides were treated with 4% (v/v) formaldehyde in 2× SSC for 10 min and washed 5 min in 2× SSC (3 times), then dehydrated in ethanol series 70%, 90% and 96% for 2 min in each at the room temperature. The denatured probe mixture was dropped onto dry slides and covered with a coverslip. Chromosome specimens were denatured by heating at 74°C for 2 min, and hybridization was performed in a moist chamber at 37°C for 24 h. After washing in the 2× SSC at 58°C for 20 min, slides were dehydrated in ethanol series 70%, 90% and 96% for 2 min in each, air-dried and mounted with Vectashield (Vector Laboratories, USA) containing 4’,6-diamidino-2-phenylindole (DAPI). The FISH signals were captured with an ORCA-ER charge-coupled device camera (Hamamatsu, Japan) using an Olympus BX-61 microscope.

## Results

### X-Ray Irradiation Induces B Chromosome Aberrations

**FIGURE 1:**
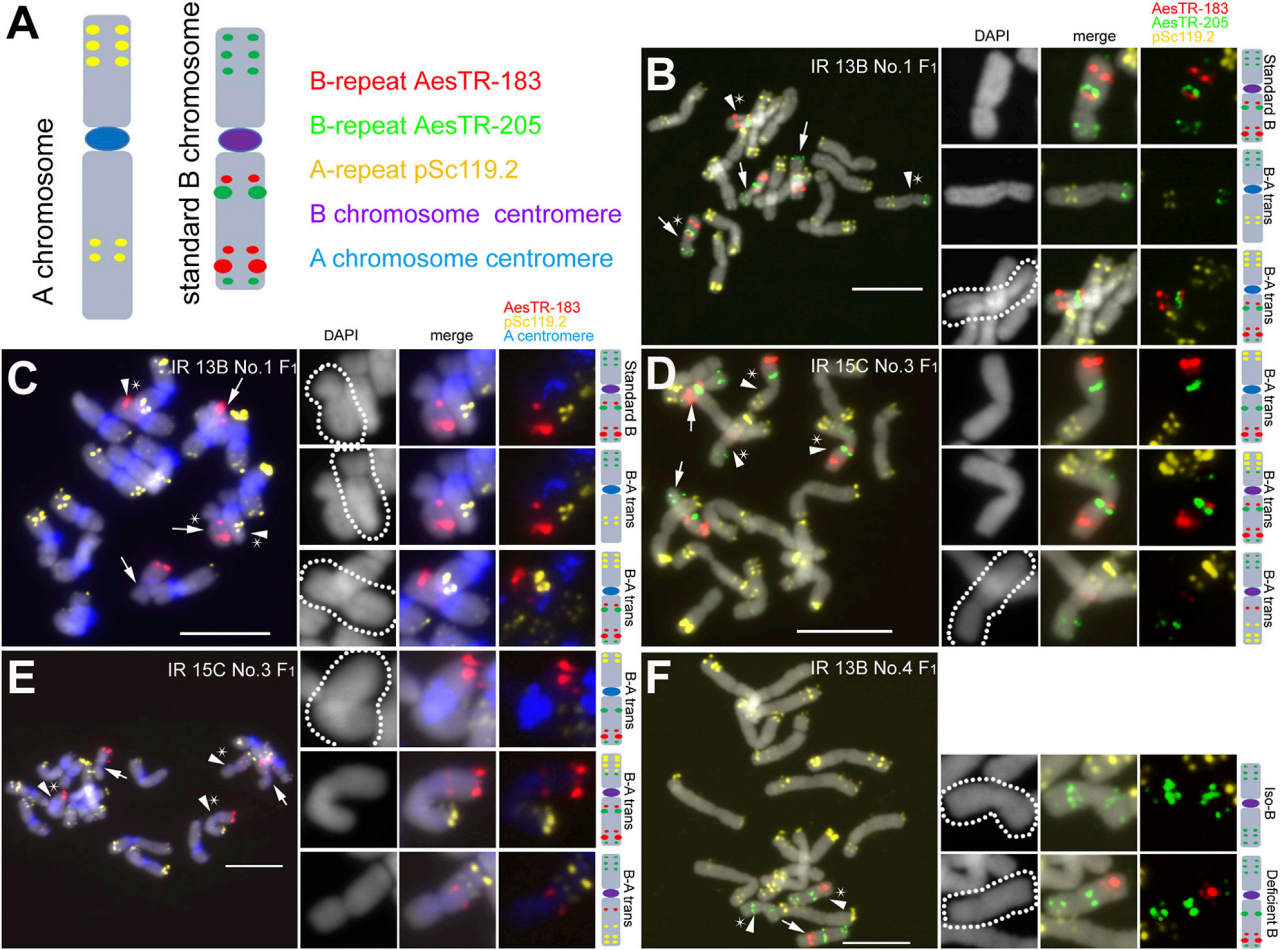
Characterization of mitotic metaphase cells isolated from shoots of *Ae. speltoides* plants possessing B chromosome variants. **(A)** Idiogram of a typical A chromosome (chromosome 7S) showing the position of A chromosome-specific repeat pSc119.2 (in yellow) and a standard B chromosome showing the position of the B chromosome-specific repeats AesTR-183 (in red) and AesTR-205 (in green). The A chromosome (peri)centromere-specific probe pBs301 (in blue) allowed the identification of the centromere origin. **(B,C)** F_1_ plant IR 13B No.1 carries 3 standard Bs and two B-A translocations possessing A chromosome centromeres. **(D,E)** F_1_ plant IR 15C No.3 carries two standard Bs and 3 B-A translocations. Two B-A translocations contain centromeres from B, while one B-A translocation contains an A centromere. **(F)** F_1_ IR 13B No.4 contains a standard B and one short arm iso-B and B with an interstitial loss of chromatin. Standard Bs and B variants are marked with arrows and arrowheads, respectively. Stars indicate the chromosomes selected for the zoom-in view. The schemata of selected B chromosome variants show the chromosomal distribution of all applied FISH probe combinations. Scale bars, 10 μm.

To generate B chromosome variants, spikes of +2B and +3B *Ae. speltoides* plants were X-ray irradiated with a dosage of 13, 15 or 18 Gray. After that, pollen was collected and used to cross-fertilize 0B plants (Supplementary Table S1). F_1_ seeds were obtained independently of the dosage used, however only those plants survivied that were generated from pollen radiated with 13 or 15 Gray. Roots and shoots of resulting F_1_ plants and selfed progenies of subsequent generations (F_2_, F_3_) were used for FISH screening using B-specific (AesTR-183, AesTR-205), and A-specific (pSc119.2) FISH probes (Figure 1A). At least three shoots and roots per +B plant were investigated. 8 of 14 F_1_ +B plants revealed in all analyzed shoots in addition to standard B chromosomes modified Bs like seven types of B-A translocations, three types of deficient Bs, and one type of short arm isochromosome (Table 1). B-A translocations were only observed in the shoots of 5 of 14 of F_1_ plants.

**TABLE 1:** Identified B chromosome variants in F_1_ to F_3_ generations of *Ae. speltoides* plants.

B chromosome variants		In 14 F_1_ plants analyzed		In 44 F_2_ plants analyzed		In 50 F_3_ plants analyzed	
		Shoots n	Roots n	Shoots n	Roots n	Shoots n	Roots n
B-A translocations		7 (in 5 plants)	10 (in 6 plants)	4 (in 4 plants)	10 (in 6 plants)	2 (in 3 plants)	8 (in 4 plants)
Deficient Bs	with centromeric constriction	3 (in 4 plants)	2 (in 2 plants)	1 (in 1 plant)	11 (in 24 plants)	0	2 (in 1 plant)
	without centromeric constriction	0	1 (in 1 plant)	0	4 (in 2 plants)	0	2 (in 2 plant)
B isochromosome		1 (in 1 plant)	0	0	1 (in 1 plant)	0	0
Standard B		In 13 plants	0	In 29 plants	In 2 plants	In 16 plants	In 1 plant

n: number of B chromosome variant types observed.

The application of the A chromosome (peri)centromere-specific probe pBs301 allowed the identification of the centromere origin of the B-A translocation chromosomes. In 57.1% of F1 B-A translocations, the centromere derived from an A chromosome (Table 2). For example, besides three standard Bs, two types of B-A translocations were found in the shoots of the genotype IR 13B No.1 (F_1_) (Figure 1B). One translocation occurred between the long B arm and the short arm of an A chromosome. Another translocation happened between the B short arm and an A long arm. Both translocation chromosomes possessed the A centromere (Figures 1B,C). Three different types of B-A translocations were detected in the shoots of IR 15C No.3 (F_1_). In two B-A translocations, the centromere originated from the B, while the centromere of another B-A translocation derived from an A chromosome (Figures 1D,E). In the shoots of IR 13B No.4 (F_1_), a rearrangement of the B occurred with loss of the AesTR-183 signals near the centromeric region. In addition, a short arm iso-B was found (Figure 1F). The iso-B chromosome may have been generated after the fusion of two B short arms.

**TABLE 2:** A or B chromosome-derived centromere origin in B-A translocations.

Generation	Tissue	Number of analysed B variants	A centromere origin	B centromere origin	Centromere origin not analysed
F_1_	Shoots	7	4	3	0
	Roots	10	4	0	6
F_2_	Shoots	4	3	1	0
	Roots	10	7	0	3
F_3_	Shoots	2	2	0	0
	Roots	8	8	0	0

### Mutagenesis Results in Escapees of the Root-Specific B Chromosome Elimination Process

**FIGURE 2:**
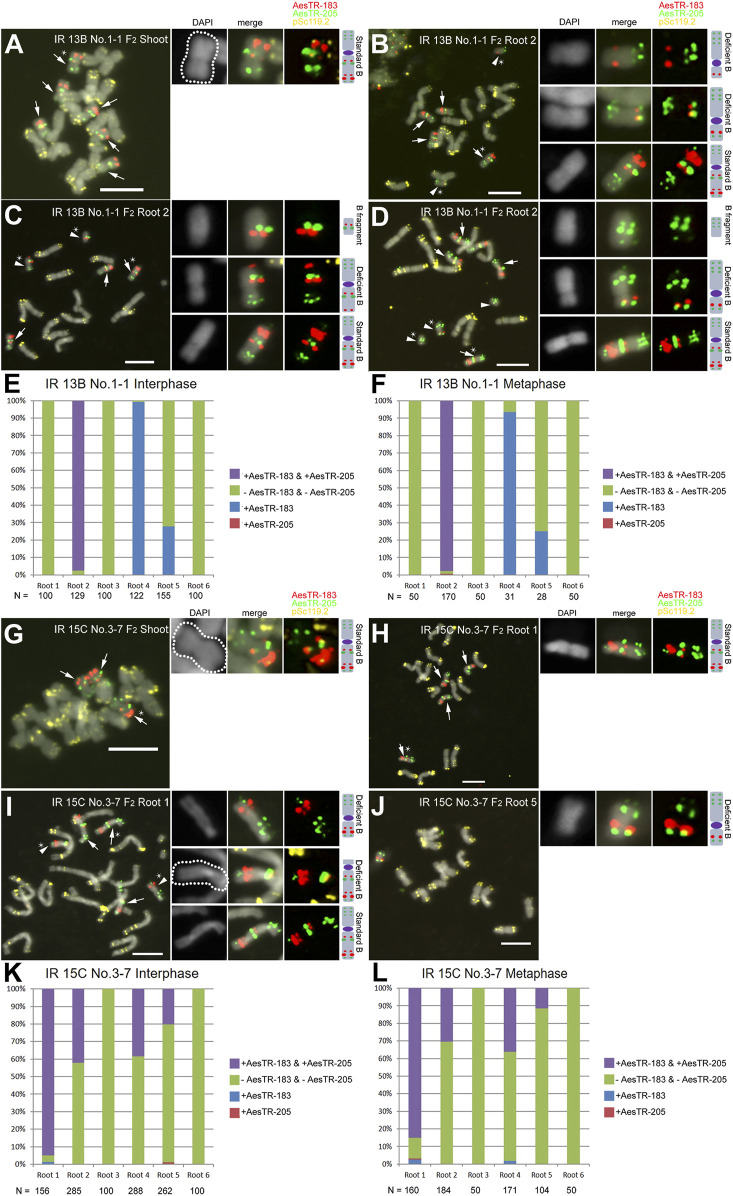
Escapees of the root-specific B chromosome elimination process. **(A)** F_2_ plant IR 13B No.1-1 carries six standard Bs in shoots. **(B–D)** Root 2 of the same plant showed standard Bs and different types of deficient Bs with or without visible centromeres. Quantification of B variants in interphase **(E,K)** and metaphase **(F,L)** root cells. **(G)** F_2_ plant IR IR 15C No.3-7 carries three standard Bs in shoots. **(H–J)** Root cells of F_2_ plant IR 15C No.3-7 shows three metaphases with standard Bs and different B varaints. Standard Bs and B variants are marked with arrows and arrowheads, respectively. Stars indicate the chromosomes selected for the zoom-in view. Scale bars, 10 μm.

In contrast to non-irradiated +B plants where Bs only exist in the aerial parts of *Ae. speltoides* plants (Mendelson and Zohary, 1972; Ruban et al., 2020), 10 of 14 mutagenized F_1_ +B plants revealed B-specific FISH signals in a subset of roots (Table 1; Supplementary Table S2) and root cells. In all shoots of IR 13B No.1-1 (F_2_), six standard Bs were identified (Figure 2A). However, only 3 of 6 roots of the same plant showed B-specific signals. Three metaphase cells of root 2 showed different B variants, including standard Bs, three types of deficient Bs with a B centromere and two types of fragments without any visible centromeric constrictions (Figures 2B–D). To quantify the frequency of root-located Bs at least 100 interphase and 28 metaphase cells were analyzed in each of the six different roots of IR 13B No.1-1 (F_2_) (Figures 2E,F). Only in one of six roots both B-specific repeats were almost consistently detected in interphase and metaphase cells, and two of six roots displayed AesTR-183 specific signals only. Cells with AesTR-205-signals alone were rarely found.

**FIGURE 3:**
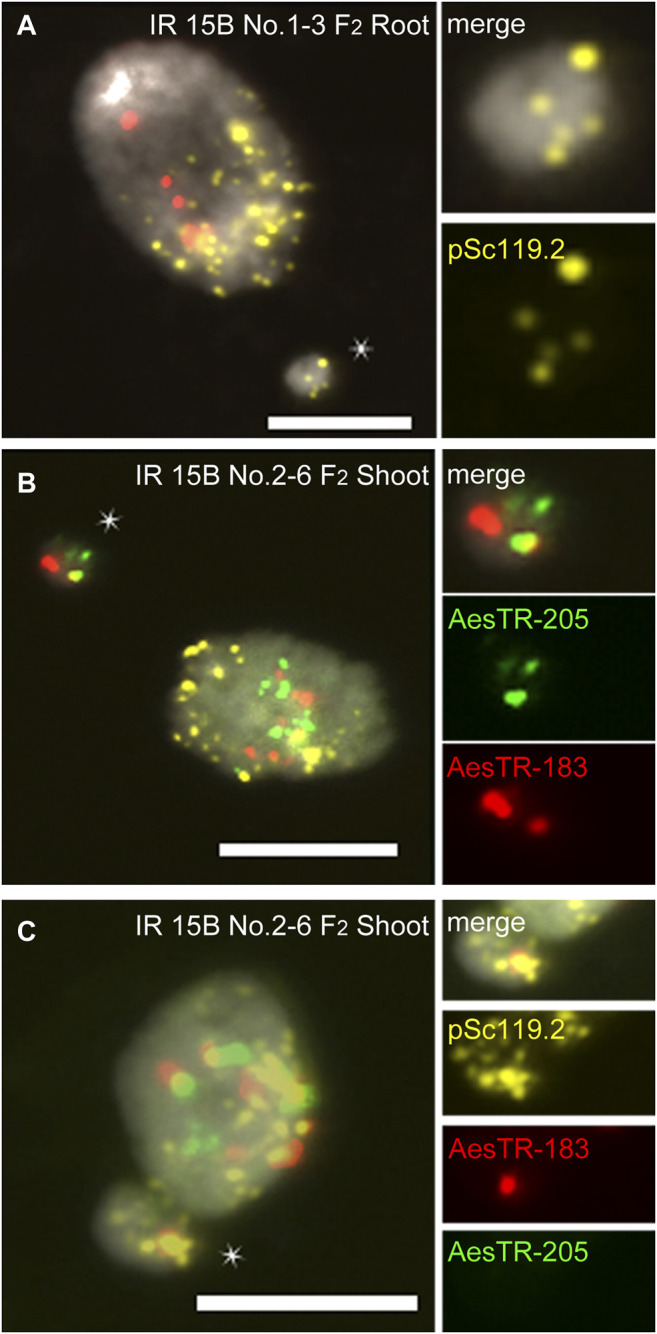
Characterization of micronuclei observed in mutagenized +B *Ae. speltoides* plants. **(A)** A chromosome containing micronucleus found in the root of IR 15B No.1-3. **(B)** B chromosome-derived micronucleus and **(C)** B-A translocation chromosome derirved micronucleus found in the shoot of IR 15B No.2-6. Micronuclei are marked by star. Scale bars, 10 μm.

In IR 15C No.3-7 (F_2_), three standard Bs were observed in all shoots (Figure 2G), but only four of six roots showed B chromosomes (Figures 2K,L). In root 1, four standard Bs were found in one cell, and three standard Bs and two types of deficient Bs in another cell (Figures 2H,I). In root 5 of the same individual, one deficient B was observed (Figure 2J). In other plants additional B variants were found, including various iso Bs, and deficient Bs with or without centromeric constictions (Supplementary Figures S1A,B). Two types of translocations between arms of Bs and As were found in one of the IR 13B No.1-3 (F_2_) roots (Supplementary Figure S1C). In the roots of IR 15B No.2-9 (F_2_), a translocation occurred resulting in a fusion of terminal fragment of the B long arm and a long A arm (Supplementary Figures S1D,E). A B-A translocation with a pair of B-specific AesTR-183 signals near the pericentromeric region of an A chromosome was detected in the root of IR 15B No.1-6-2 (F_3_) (Supplementary Figures S1F,G). Hence the B composition and frequency differ between individual roots of the same plant. Some B chromosome variants inconsistently escaped the elimination process. As a result, a B chromosome mosaicism was found in roots. In addition, independently of the proto-root cell restricted process of B chromosome elimination during embryogenesis, in the roots and shoots of F_2,3_ plants few micronuclei were found showing either A, B or both types of signals (Figure 3; Supplementary Figure S2). As micronuclei were never found in the shoots of non-irradiated plants, the formation of micronuclei might occur in response to irradiation.

### Some B-A Translocations Are Heritable Over Generations

**FIGURE 4:**
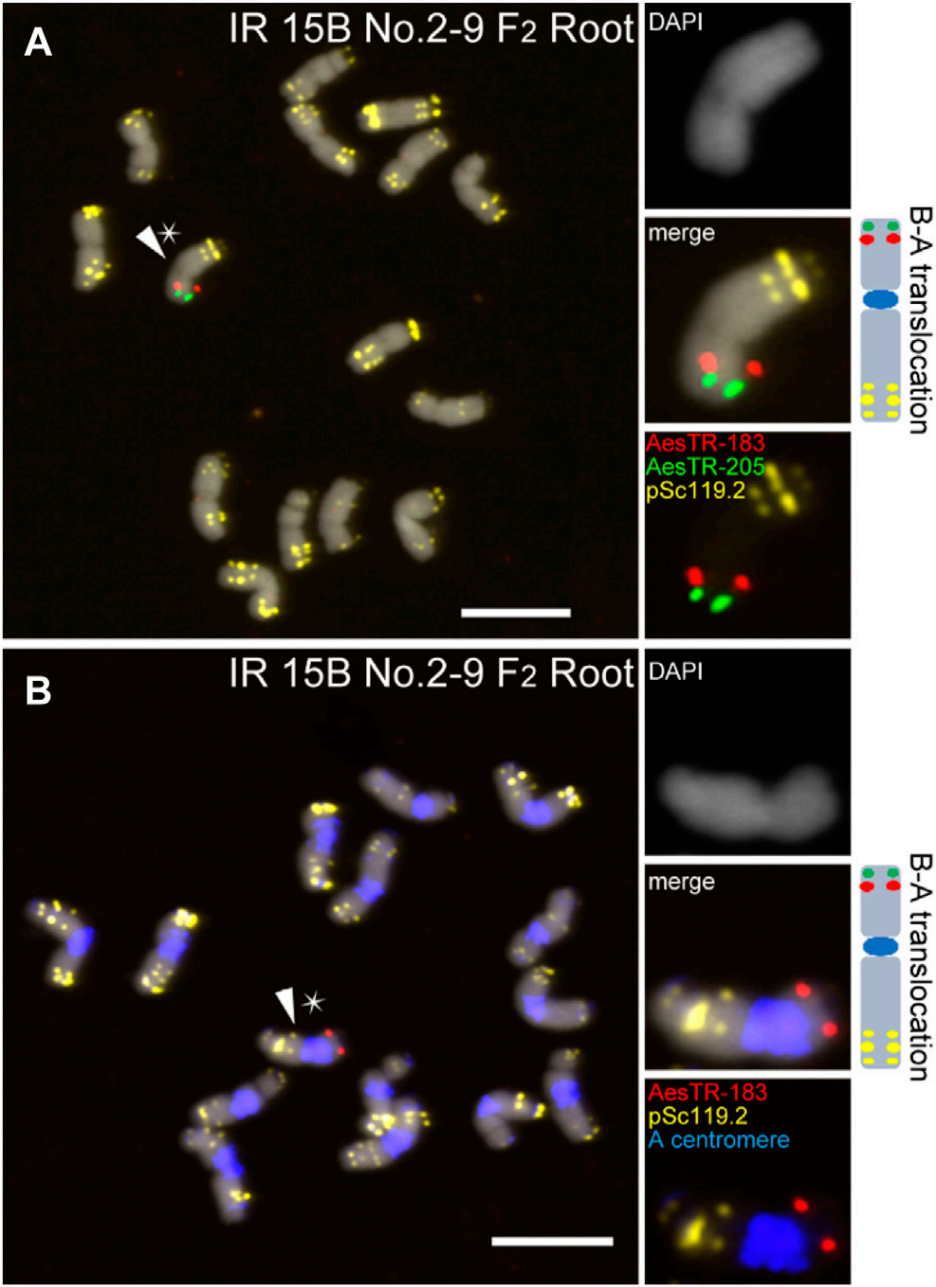
The B-A translocation chromosome of IR 15B No.2 is stable in F_2_ and F_3_ plants in root and leaf tissues. **(A)** A small part of the long B arm was fused to a broken A chromosome. **(B)** The B-A translocation carries A chromosome centromere. Stars indicate the chromosomes selected for the zoom-in view. The schemata of selected B chromosome variants show the chromosomal distribution of all applied FISH probe combinations. Scale bars, 10 μm.

In general, the frequency of B-A translocations decreased over generations from 35.7% to 6% in shoots and 42.9% and 8% in roots in F_1_ and F_3_ plants, respectively (Table 1). For example, in the shoots of IR 15B No.1 two stable B-A translocations with whole long/short arms translocated between the B chromosome and an A chromosome carrying A centromeres were identified in the F_2_ and F_3_ progeny (Supplementary Figures S3A–F). In this translocation chromosome, three pairs of pSc119.2 signals in the short arm and two pSc119.2 clusters in the long arm were detected. However, in the roots of IR 15B No.1, only one type B-A translocation was found. This is likely masked by the small number of roots investigated considering the random occurrence of B rearrangements in roots (Supplementary Figure S3G). In the offspring, some of the B-A translocations were the same in roots and shoots, but we also found root and shoot restricted B-A translocations (Supplementary Figures S3H–N). Interestingly, the B-A translocation chromosome of IR 15B No.2 is stable in F_2_ and F_3_ plants in root and leaf tissues (Figure 4; Supplementary Figure S4). Here a small part of the long B chromosome arm was fused to an A chromosome. A summary of all observed B chromosome variants in F_1_–F_3_ is shown in Supplementary Figure S5.

## Discussion

B chromosomes are supernumerary and widely present in eukaryotes. Interestingly, previous studies showed numerous B chromosome variants in natural *Ae. speltoides* genotypes (Ruban et al., 2014; Shams and Raskina, 2020). Naturally occurring Bs are stably present in the cells of the aerial organs, but they are absent in the roots because of B chromosomal elimination upon the onset of embryo differentiation (Mendelson and Zohary, 1972; Ruban et al., 2020). Although it remains open why the root-specific elimination process occurs, it is proposed that B chromosome located root-specifically expressed genes may be deleterious for plant development. Alternatively, the elimination process is a byproduct of selection for B chromosome maintenance in shoot tissue (Ruban et al., 2020). Here, we show that some B chromosome variants can inconsistently escape the elimination process in *Ae. speltoides* roots.

We produced and characterized numerous B-A translocations, B chromosome deletions, and B-isochromosomes by the application of irradiated +B pollen for crossing and a FISH-based screening of F_1_ plants and their two subsequent generations. 5 of 46 rearranged Bs (3 of 5 are B-A translocations) not only occurred in shoots but also in some roots cells of *Ae. speltoides*. Thus some B-A translocations possessing an A chromosome centromere escaped at least partially the elimination process during early embryogenesis over generations. Although yet unknown, it is plausible that mutagenesis may have altered or eliminated B and/or A-located sequences, controlling the process of chromosome elimination in roots.

Intriguingly, in the same individuals, the B variants were always stable in all analyzed shoot cells; however, they showed variation in root cells among individuals. Even though we found the same B-A translocations and B variants in roots like in shoots, we observed additional types of B variants in the roots. None *de novo* formed B-A translocation was found in the shoots of offspring plants. Thus, a root restricted restructuring of Bs occurred, likely due to dysfunction or loss of genes related to the B chromosome elimination process.

Some B-A translocations were stable across two generations in shoots. In the shoots of the F_2_ generation, we found four types of B-A translocations, and three of them possessed an A chromosome-derived centromere. It is likely that the stable inheritance of B-A translocations is associated with the A centromere origin of the translocation chromosomes. On the other hand, the absence of root-located B centromere possessing B-A translocations implies that the centromeric region of the B is an essential component of the chromosome elimination process.

In maize, B-A translocations are often maintained in the heterozygous status by crossing as female (Roman, 1947; Beckett, 1978). In rye, none of the *de novo* generated B-A translocations was transmitted to the progeny (Hasterok et al., 2002). These observations may indicate a species- or an A chromosome type-specific tolerance for B-A translocations. In *Ae. speltoides* only the B-A translocation chromosome of IR 15B No.2 is stable in F_2_ and F_3_ plants in root and leaf tissue. Here a small B chromosome arm fragment was fused to an A chromosome. This B fragment was maintained, likely due to its small size and neutral character. However, none of the other B chromosome variants was stable in roots across three generations. Thus the generation and analysis of a larger population of mutagenized +B plants will be required to identify beside the B chromosome centromere additional chromosome regions involved in the regulation of B chromosome elimination.

## Data Availability

The original contributions presented in the study are included in the article/Supplementary Material, further inquiries can be directed to the corresponding author.
