# Supplementary material for: B-A Chromosome Translocations Possessing an A Centromere Partly Overcome the Root-Restricted Process of Chromosome Elimination in Aegilops speltoides
Source: Front Cell Dev Biol. 2022 Mar 28;10:875523. doi: 10.3389/fcell.2022.875523 (PMC8995527; doi:10.3389/fcell.2022.875523)
Supplement: Supplementary file 1 [file Table1.docx]

**Supplementary Table 1.** Plant material used in this study. The spikes from plants carrying Bs were X-ray irradiated with doses at 13, 15 and 18 Gray shortly before anthesis.

| Genotype | Cross combination | Radiation dose | Obtained seeds/survived plants | |
| --- | --- | --- | --- | --- |
| IR 13A | P33-7 (0B) x P42f1-33spike4 (2 or 3 Bs) | 13 Gray | 3 F_1_ seeds, 1 plant |  |
| IR 13B | P33-5 (0B) x P42f1-33spike5 (2 or 3 Bs) | 13 Gray | 8 F_1_ seeds, 5 plants |  |
| IR 13C | P33-1 (0B) x P42f1-33spike5 (2 or 3 Bs) | 13 Gray | 4 F_1_ seeds, 2 plants |  |
| IR 13D | P33-8 (0B) x P42f1-33spike5 (2 or 3 Bs) | 13 Gray | 4 F_1_ seeds, 2 plants |  |
| IR 15A | P33-5 (0B) x P42f1-1spike1 (2 Bs) | 15 Gray | 6 seeds |  |
| IR 15B | P33-8 (0B) x P42f1-10spike2 (3 Bs) | 15 Gray | 6 F_1_ seeds, 2 plants |  |
| IR 15C | P33-7 (0B) x P42f1-1spike3 (2 Bs) | 15 Gray | 7 F_1_ seeds, 4 plants |  |
| IR 15D | P33-4 (0B) x P42f1-10spike1 (3 Bs) | 15 Gray | 2 F_1_ seeds |  |
| IR 15E | P33-2 (0B) x P42f1-1spikes1-2 (2 Bs) | 15 Gray | 1 F_1_ seeds |  |
| IR 18A | P33-5 (0B) x P42f1-33spike2 (2 or 3 Bs) | 18 Gray | 3 F_1_ seeds |  |
