# Supplementary figures and images for: B-A Chromosome Translocations Possessing an A Centromere Partly Overcome the Root-Restricted Process of Chromosome Elimination in Aegilops speltoides
Source: Front Cell Dev Biol. 2022 Mar 28;10:875523. doi: 10.3389/fcell.2022.875523 (PMC8995527; doi:10.3389/fcell.2022.875523)

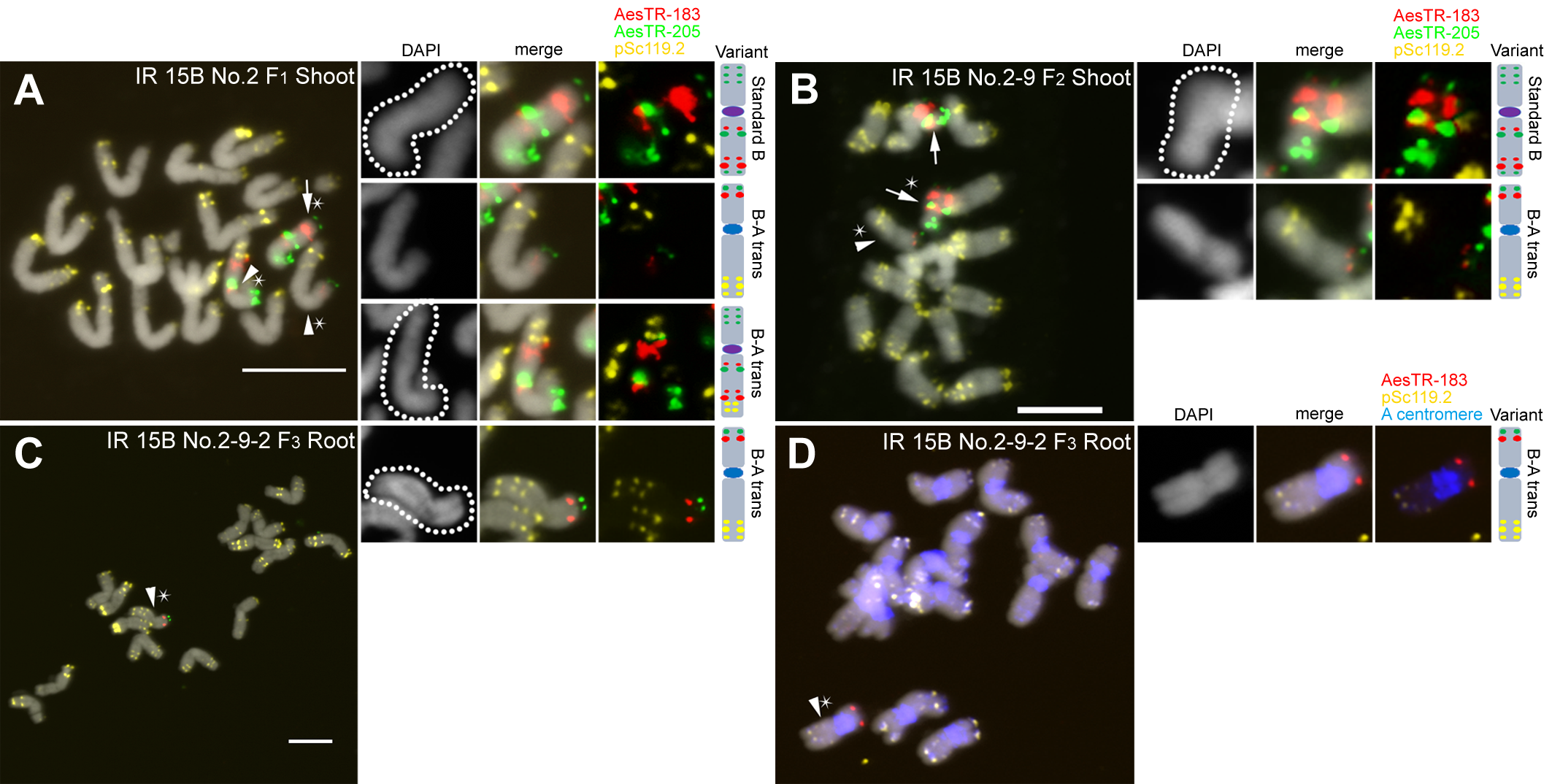

Supplement: Supplementary file 2 [file Image3.TIF]

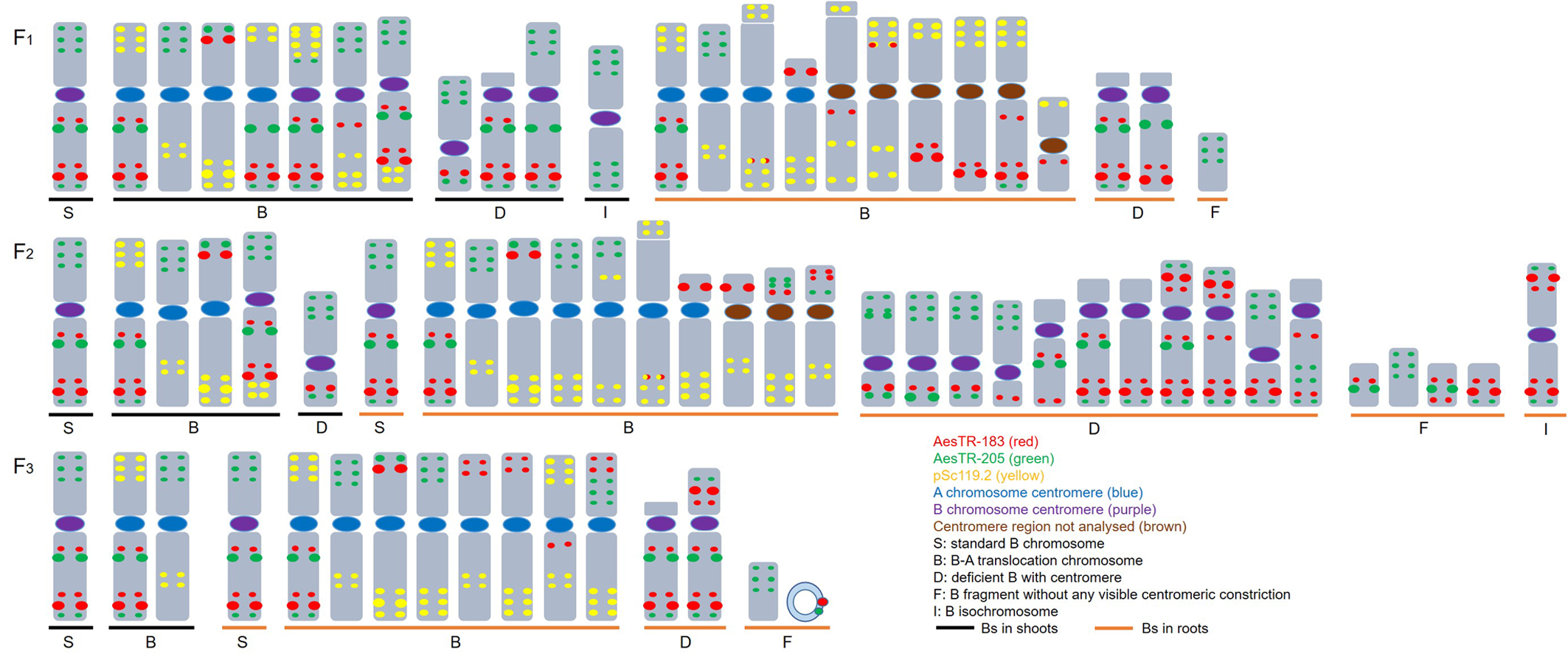

Supplement: Supplementary file 3 [file Image4.TIF]

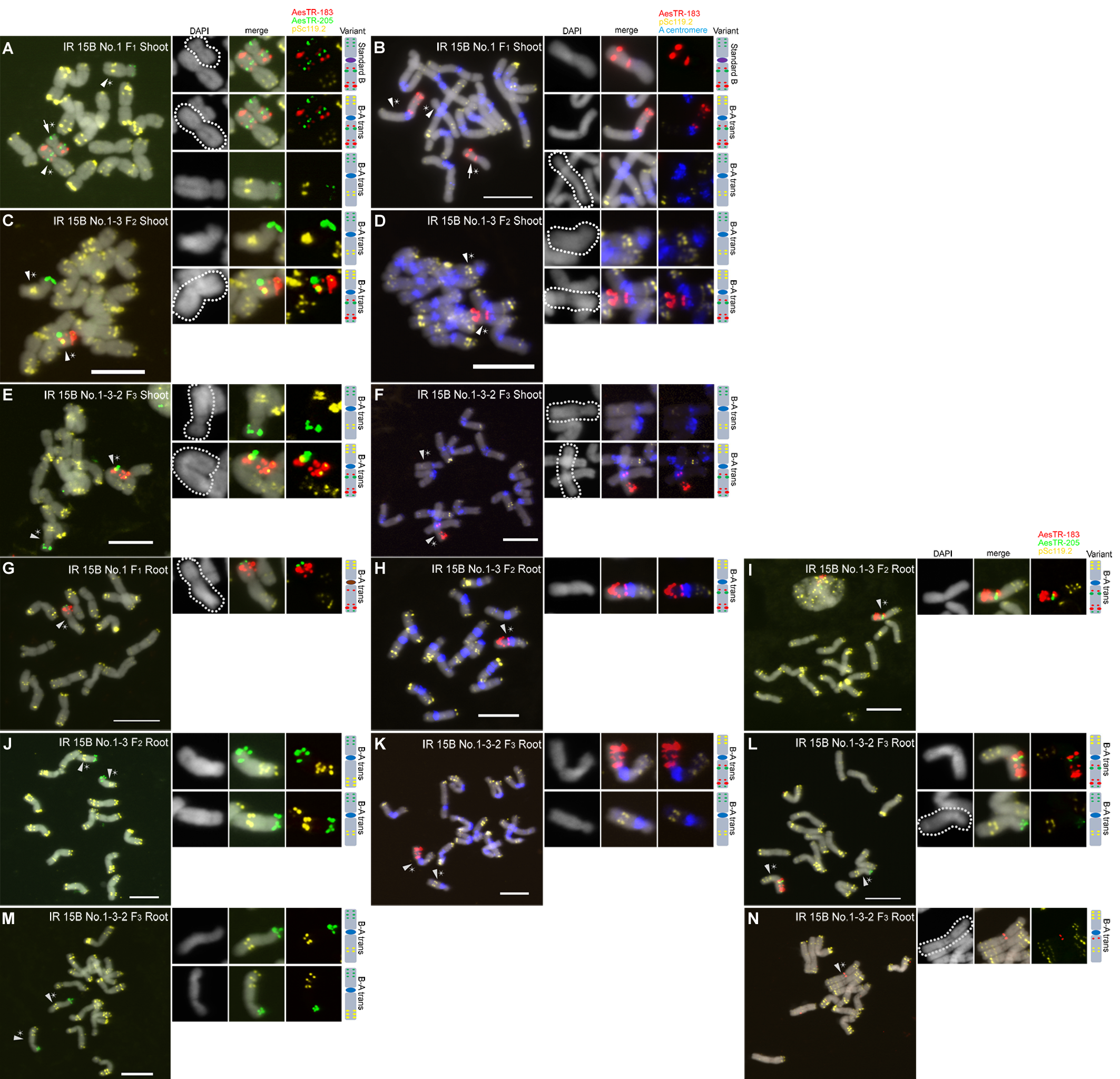

Supplement: Supplementary file 4 [file Image2.TIF]

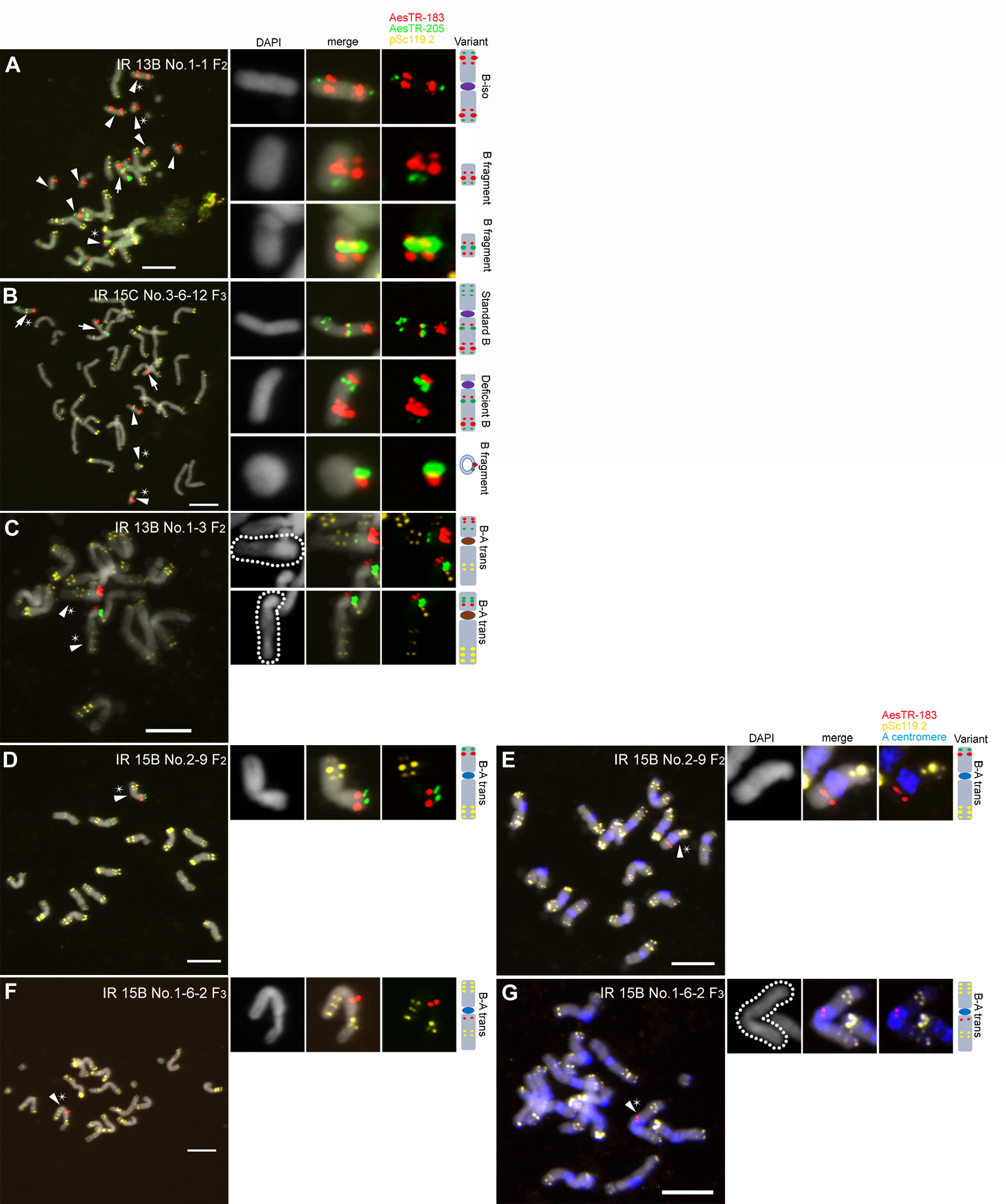

Supplement: Supplementary file 5 [file Image1.TIF]

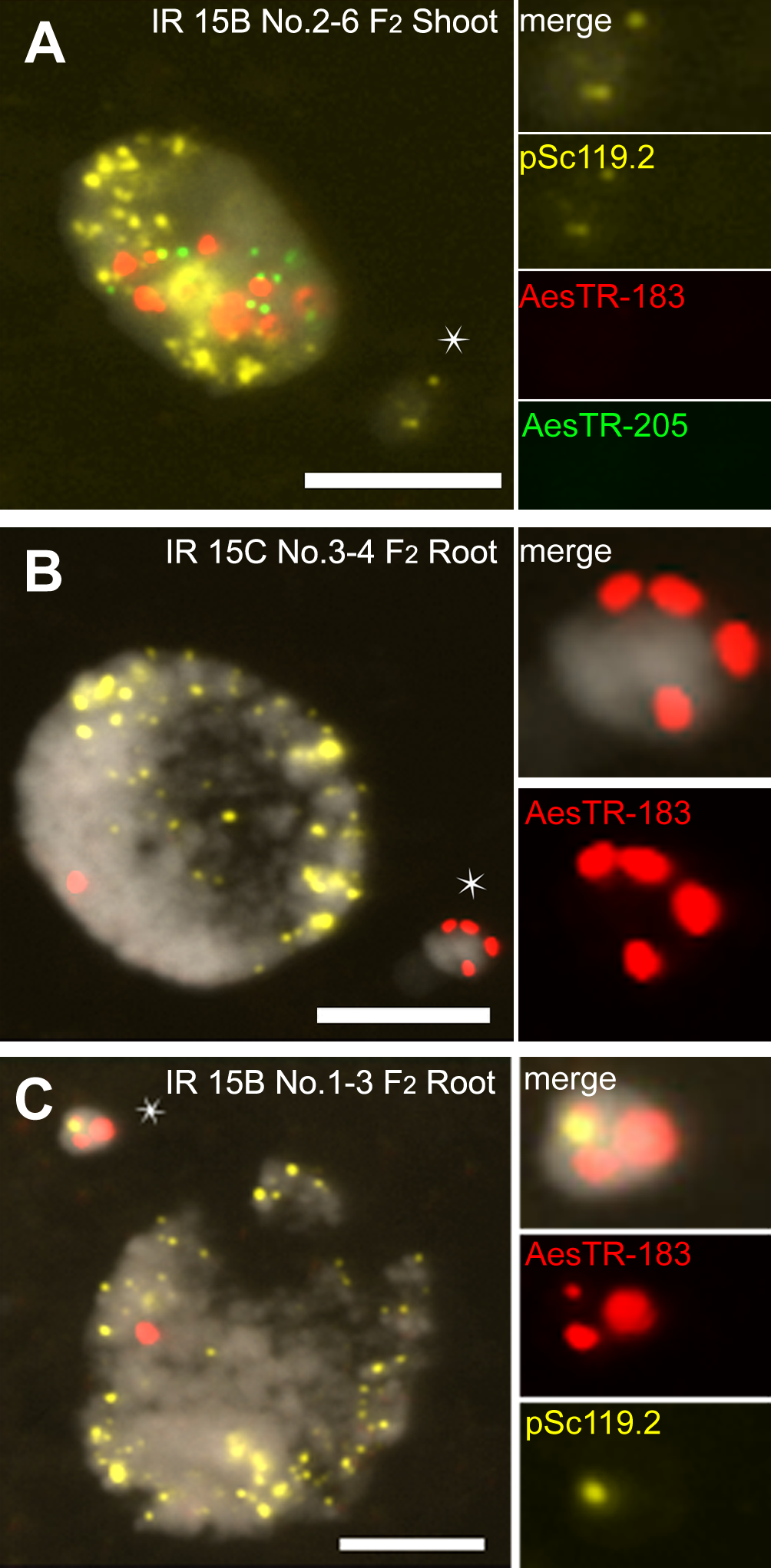

Supplement: Supplementary file 7 [file Image5.TIF]
