# Supplementary material for: B-A Chromosome Translocations Possessing an A Centromere Partly Overcome the Root-Restricted Process of Chromosome Elimination in Aegilops speltoides
Source: Front Cell Dev Biol. 2022 Mar 28;10:875523. doi: 10.3389/fcell.2022.875523 (PMC8995527; doi:10.3389/fcell.2022.875523)
Supplement: Supplementary file 6 [file Table2.docx]

**Supplementary Table 2.** Plant material from F_1_ to F_3_ analyzed in this study.

| F1 (14 plants) | shoot | root | F2 (44 plants) | shoot | root | F3 (50 plants) | shoot | root |
| --- | --- | --- | --- | --- | --- | --- | --- | --- |
| IR 13A No.1 | + | + | IR 13B No1-1 | + | + | IR 13B No.3-3-1 |  |  |
| IR 13B No.1 | + | + | IR 13B No1-2 |  |  | IR 13B No.3-3-2 |  |  |
| IR 13B No.2 | + | + | IR 13B No1-3 | + | + | IR 113B No.3-3-3 |  |  |
| IR 13B No.3 | + | + | IR 13B No1-4 | + |  | IR 13B No.3-3-4 |  |  |
| IR 13B No.4 | + | + | IR 13B No1-5 | + |  | IR 13B No.3-3-5 |  |  |
| IR 13B No.5 | + |  | IR 13B No1-6 | + |  | IR 13B No.3-3-6 |  |  |
| IR 13C No.1 | + |  | IR 13B No1-7 | + |  | IR 13B No.3-3-7 |  |  |
| IR 13D No.1 | + | + | IR 13B No1-8 | + |  | IR 13B No.3-3-8 |  |  |
| IR 13D No.2 | + | + | IR 13B No1-9 | + |  | IR 13B No.3-3-9 |  |  |
| IR 15B No.1 | + | + | IR 13B No1-10 |  |  | IR 13B No.3-5-1 |  |  |
| IR 15B No.2 | + | + | IR 13B No3-1 | + |  | IR 13B No.3-5-2 |  |  |
| IR 15C No.1 | + | + | IR 13B No3-2 |  |  | IR 13B No.3-5-3 |  |  |
| IR 15C No.2 | + |  | IR 13B No3-3 |  |  | IR 13B No.3-5-4 |  |  |
| IR 15C No.3 | + |  | IR 13B No3-4 | + |  | IR 13B No.3-5-5 |  |  |
| In total | 14 plants | 10 plants | IR 13B No3-5 |  |  | IR 13D No.1-2-1 |  |  |
|  |  |  | IR 15B No1-1 | + |  | IR 13D No.1-2-2 |  |  |
|  |  |  | IR 15B No1-2 | + |  | IR 13D No.1-2-3 |  |  |
|  |  |  | IR 15B No1-3 | + | + | IR 13D No.1-2-4 |  |  |
|  |  |  | IR 15B No1-4 |  |  | IR 13D No.1-2-5 |  |  |
|  |  |  | IR 15B No1-5 |  |  | IR 13D No.1-2-6 |  |  |
|  |  |  | IR 15B No1-6 | + | + | IR 13D No.1-2-7 |  |  |
|  |  |  | IR 15B No1-7 | + |  | IR 13D No.1-2-8 |  |  |
|  |  |  | IR 15B No1-8 | + |  | IR 13D No.1-2-9 |  |  |
|  |  |  | IR 15B No2-1 | + |  | IR 13D No.1-2-10 |  |  |
|  |  |  | IR 15B No2-2 | + |  | IR 15B No.1-3-1 |  |  |
|  |  |  | IR 15B No2-3 |  |  | IR 15B No.1-3-2 | + | + |
|  |  |  | IR 15B No2-4 |  |  | IR 15B No.1-3-3 |  |  |
|  |  |  | IR 15B No2-5 |  |  | IR 15B No.1-6-1 |  |  |
|  |  |  | IR 15B No2-6 | + |  | IR 15B No.1-6-2 | + | + |
|  |  |  | IR 15B No2-7 |  |  | IR 15B No.1-6-3 |  |  |
|  |  |  | IR 15B No2-8 | + |  | IR 15B No.1-6-4 |  |  |
|  |  |  | IR 15B No2-9 | + | + | IR 15B No.1-6-5 |  |  |
|  |  |  | IR 15C No3-1 | + |  | IR 15B No.1-6-6 |  |  |
|  |  |  | IR 15C No3-2 | + |  | IR 15B No.2-9-1 |  |  |
|  |  |  | IR 15C No3-3 | + |  | IR 15B No.2-9-2 | + | + |
|  |  |  | IR 15C No3-4 | + | + | IR 15B No.2-9-3 |  |  |
|  |  |  | IR 15C No3-5 | + |  | IR 15C No.3-6-1 | + |  |
|  |  |  | IR 15C No3-6 | + | + | IR 15C No.3-6-2 | + |  |
|  |  |  | IR 15C No3-7 | + | + | IR 15C No.3-6-3 | + |  |
|  |  |  | IR 15C No3-8 | + |  | IR 15C No.3-6-4 | + |  |
|  |  |  | IR 15C No3-9 |  |  | IR 15C No.3-6-5 | + |  |
|  |  |  | IR 13D No1-1 | + |  | IR 15C No.3-6-6 | + |  |
|  |  |  | IR 13D No1-2 | + |  | IR 15C No.3-6-7 | + |  |
|  |  |  | IR 13D No1-3 | + |  | IR 15C No.3-6-8 | + |  |
|  |  |  | In total | 32 plants | 8 plants | IR 15C No.3-6-9 | + |  |
|  |  |  |  |  |  | IR 15C No.3-6-10 | + |  |
|  |  |  |  |  |  | IR 15C No.3-6-11 | + |  |
|  |  |  |  |  |  | IR 15C No.3-6-12 | + | + |
|  |  |  |  |  |  | IR 15C No.3-6-13 | + |  |
|  |  |  |  |  |  | IR 15C No.3-6-14 | + |  |
|  |  |  |  |  |  | IR 15C No.3-6-15 | + |  |
|  |  |  |  |  |  | IR 15C No.3-6-16 | + |  |
|  |  |  |  |  |  | IR 15C No.3-6-17 |  |  |
|  |  |  |  |  |  | In total | 19 plants | 4 plants |

+: plant revealed B-specific FISH signals.
